# Pervasive 3′-UTR Isoform Switches During Mouse Oocyte Maturation

**DOI:** 10.3389/fmolb.2021.727614

**Published:** 2021-10-18

**Authors:** Yuanlin He, Qiuzhen Chen, Jing Zhang, Jing Yu, Meng Xia, Xi Wang

**Affiliations:** ^1^ State Key Laboratory of Reproductive Medicine, Nanjing Medical University, Nanjing, China; ^2^ Department of Epidemiology, Center for Global Health, School of Public Health, Nanjing Medical University, Nanjing, China; ^3^ Key Laboratory of Cell Differentiation and Apoptosis of Chinese Ministry of Education, Shanghai Jiao Tong University School of Medicine, Shanghai, China; ^4^ State Key Laboratory of Reproductive Medicine, Clinical Center of Reproductive Medicine, First Affiliated Hospital, Nanjing Medical University, Nanjing, China

**Keywords:** oocyte maturation, isoform switches, alternative 3′-UTR isoforms, alternative polyadenylation, single oocyte RNA-seq

## Abstract

Oocyte maturation is the foundation for developing healthy individuals of mammals. Upon germinal vesicle breakdown, oocyte meiosis resumes and the synthesis of new transcripts ceases. To quantitatively profile the transcriptomic dynamics after meiotic resumption throughout the oocyte maturation, we generated transcriptome sequencing data with individual mouse oocytes at three main developmental stages: germinal vesicle (GV), metaphase I (MI), and metaphase II (MII). When clustering the sequenced oocytes, results showed that isoform-level expression analysis outperformed gene-level analysis, indicating isoform expression provided extra information that was useful in distinguishing oocyte stages. Comparing transcriptomes of the oocytes at the GV stage and the MII stage, in addition to identification of differentially expressed genes (DEGs), we detected many differentially expressed transcripts (DETs), some of which came from genes that were not identified as DEGs. When breaking down the isoform-level changes into alternative RNA processing events, we found the main source of isoform composition changes was the alternative usage of polyadenylation sites. With detailed analysis focusing on the alternative usage of 3′-UTR isoforms, we identified, out of 3,810 tested genes, 512 (13.7%) exhibiting significant switches of 3′-UTR isoforms during the process of moues oocyte maturation. Altogether, our data and analyses suggest the importance of examining isoform abundance changes during oocyte maturation, and further investigation of the pervasive 3′-UTR isoform switches in the transition may deepen our understanding on the molecular mechanisms underlying mammalian early development.

## Introduction

The proper development of mammalian oocytes is crucial for females to provide high-quality eggs for the generation of new individuals, and improper oocyte maturation usually contributes to female infertility or unhealthy early embryos (Jose-Miller et al., 2007). Oocyte maturation is the last stages of oocyte development, which typically refers to the dynamic process since the first meiotic resumption starting at oocyte germinal-vesicle breakdown (GVBD) (Pan and Li, 2019). Thereafter, oocytes go through metaphase I (MII) and then metaphase II (MII), during which oocyte meiosis is arrested again. The meiosis resumes for the second time at fertilization, and only until this point oocyte maturation is complete (Sen and Caiazza, 2013).

To disentangle the molecular mechanisms during the dynamic process of oocyte maturation, accumulating efforts were attempted from various aspects, including epigenetic regulation (He et al., 2021a), gene expression alteration (Zhao et al., 2020), protein metabolic patterns (Li et al., 2020) as well as their combinations (Gu et al., 2019). It has been shown in literature that the chromatin modification and remodeling in oocytes at the germinal vesicle (GV) stage are largely related to global transcriptional silence before entering meiotic resumption (De La Fuente, 2006). As the pause of transcription continues until zygotic genome activation post fertilization (Bouniol-Baly et al., 1999; Ma et al., 2001), the overall amount of RNA transcripts in oocytes gradually declines, though different genes degrading at various speeds (Zhao et al., 2020). For example, genes related to meiosis I cell cycle process and mitochondrion organization degraded at the top speed during the GV to MII transition in mice (Zhao et al., 2020). With the degradation of such no-longer-needed transcripts, oocytes progressively get ready for fertilization and the sequential early embryonic development (Wu and Dean, 2020).

Recently, researchers have revealed that the RNA degradation machinery is important to alter the transcriptome conformation for developmental processes (Belair et al., 2019; Slobodin et al., 2020; Wu and Dean, 2020), and its regulation is largely dependent on poly(A) tails (Slobodin et al., 2020) and related to 3′-UTR isoforms (Zheng et al., 2018). Although gene-level transcriptomic changes during oocyte maturation have been largely depicted, such changes at the isoform level remain elusive, which would likely impede our advanced understanding in the regulatory mechanisms in RNA degradation and also the functional consequence from fast turnover of selected transcripts. In this study, we focused on the isoform-level transcriptomic differences among main stages of mouse oocyte maturation, demonstrating that the isoform-level changes provided additional information for improving oocyte clustering with respect to their stages. More importantly, we identified 512 genes exhibiting significant changes in 3′-UTR isoform composition between oocytes at the GV and the MII stages, which were linked to essential cellular functions important for the oocyte maturation.

## Materials and Methods

### Mice

All animal maintenance and experimental procedures were carried out according to guideline of the Animal Experiment Administration Committee of Nanjing Medical University, Jiangsu, China. We used C57BL/6 females to collect oocytes. All mice were given access to food and water, and were maintained on a 12:12 h light-dark artificial lighting cycle, with lights off at 7 p.m.

### Collection of Mouse Oocytes

C57BL/6 female mice (6–8 weeks old) were intraperitoneally injected with pregnant mare serum gonadotropin (PMSG, 5 IU, Ningbo Sansheng Pharmaceutical Co., ltd., P.R China), followed by human chorionic gonadotropin (hCG, 5 IU, Ningbo Sansheng Pharmaceutical Co., ltd., P.R China) 48 h later. GV oocytes were isolated from the ovaries 48 h after PMSG injection, and MII oocytes were collected from the ampulla 15 h after hCG injection. For MI oocytes, nude oocytes with clear GVs were cultured in M16 medium in 5% CO2 at 37°C and harvested at 8 h of culture for subsequent analysis as previously described (Jin et al., 2019). All oocytes were collected in hyaluronidase-containing M2 medium (Millipore) drops. Oocytes were selected with morphology, with zona pellucida gently removed by treatment of Acidic Tyrode’s solution (Sigma, cat. no. T1788) and polar body removed by gently pipetting with glass pipette. Thereafter, oocytes were washed with 0.1% BSA/PBS, and transferred into 0.2 ml tubes with the lysis buffer for RNA-seq.

### Single Oocyte RNA-Seq Library Preparation and Sequencing

After washed three times with 0.1% BSA/PBS, each oocyte was then transferred into 0.2 ml PCR tubes containing 2 μl lysis buffer. Transcriptome libraries were prepared following the Smart-seq2 protocol. Sequencing libraries were constructed by using KAPA HyperPlus Kit (Kapa Biosystems) according to the manufacturer’s instructions. All libraries were sequenced on the Illumina NovaSeq 6,000 platform with 150-bp paired-end mode at the Annoroad Genomics Company (Beijing, China).

### Processing of Single Oocyte RNA-Seq Data

The quality-control metrics of the single oocyte RNA-seq data was checked by *FastQC* (v0.11.9) (Brown et al., 2017). According to its result, we used *Trim Galore* (v0.6.4) to trim adapters from reads and remove low quality bases in the data, with arguments length >= 25, stringency = 3 and quality = 25. Then the trimmed reads were then mapped by *STAR* (v2.7.6a) (Dobin et al., 2013) against the mouse reference genome (mm10) and its corresponding GENCODE gene annotation (VM20). In this process, we also added the information of ERCC spike-ins to the reference. Thereafter, *HTSeq* (Anders et al., 2015) (v0.12.4) was used to count the reads aligned to each gene for gene-level expression quantification. In parallel, we also used *RSEM* (Li and Dewey, 2011) (v1.3.1) to align and calculate the expression at both the gene level and the transcript level.

### Batch Correction and Cell Clustering

Among a few tested tools, we chose *Combat* (Zhang et al., 2020) function in the *sva* (Zhang et al., 2020) (v3.38.0) R package for batch-effect correction according to their performance. In order to prevent overfitting, we used the stage information as the input of the *mod* parameter. Taking the batch-corrected expression matrix as the input, we used *Seurat* (Stuart et al., 2019) (v3.2.3) R package for general single oocyte RNA-seq data analysis. In brief, the data were first normalized by the *NormalizeData* function and the top 2000 highly variable genes (HVG) were selected as the feature genes downstream analysis. Principal component analysis (PCA) was performed using the *RunPCA* function, and the first five principal components (PCs) were retained for clustering analysis with the *FindClusters* function in the *Seurat* package.

### Analysis of Differential Expression at Gene and Isoform Levels

Due to the batch-effect issues in our data, we only considered the six GV-stage oocytes and the six MII-stage oocytes for differential expression analysis. The *edgeR* (Robinson et al., 2010) (v3.26.8) and *DESeq2* (Love et al., 2014) (v1.30.1) package was used to identify differentially expressed genes (DEGs) and differentially expressed transcripts (DETs) between the GV and MII oocytes. To achieve this, we took the gene expression matrices calculated by *HTSeq* and *RSEM*, and the transcript expression matrix calculated by *RSEM* as the input of *edgeR* or *DESeq2*. Genes or transcripts with false discovery rate (FDR) < 0.05 and expression fold changes >2 were considered as DEGs or DETs. Gene Ontology (GO) enrichment analysis of DEGs and DETs was performed with DAVID (Huang da et al., 2009) (v6.8).

### Analysis of Alternative Splicing

*SUPPA2* (Trincado et al., 2018) (v2.3) identifies seven types of alternative splicing (AS) events, including skipping exons (SE), alternative 5′ or 3′ splice sites (A5/A3), retained intron (RI), mutually exclusive exons (MX), and alternative first or last exons (AF/AL). GENCODE (VM20) annotation was used as reference to generate all splicing events, and *SUPPA2* was used to quantify event inclusion levels with transcript expression (TPM) matrix obtained by *RSEM*. For each AS event, we define its existence at one stage only when at least half of the oocytes of this stage had valid PSI values (PSI ! = NA). Among the AS events co-existing at both stages, *SUPPA2* identified differentially spliced events. *rMATS* (Shen et al., 2014) (v4.1.0) identifies five types of AS events, including SE, A5, A3, RI, MX. Taking the BAM files of GV and MII oocytes as the input, differentially spliced events were also identified by *rMATS*.

### Detecting Alternative Usage of 3′-UTR Isoforms

Based on the GENCODE gene annotation, *DaPars* (Xia et al., 2014) (v0.9.1) was adopted to infer unannotated proximal alternative poly(A) sites that result in alternative 3′-UTR isoforms. For its input, we converted bam files to wiggle files by *deepTools* (Ramirez et al., 2016) (v3.5.0) and *bigWigToWig* binary downloaded from the UCSC Genome Browser. Then, *DaPars* calculated the percentage of distal poly(A) site usage index (PDUI) for each oocyte, and tested for significant alternative poly(A) site usage between stages using Fisher’s exact test. The raw *p* values were subjected to multiple testing adjustment, so that FDR values were calculated. We considered those alternative usage 3′-UTR isoforms between GV and MII oocytes as significant, only if the FDR ≤0.05, the fold change of PDUI ≥1.5, and the absolute differences of PDUI ≥0.3.

### Validation of 3′-UTR Isoform Switches

Total RNA was isolated from oocytes with the RNeasy Mini kit (Qiagen). cDNA was synthesized by using SuperScript II Reverse transcriptase (Invitrogen) with oligo-dT primer at 42°C for 90 min. PCR primers were design for the regions shared between alternative 3′-UTR isoforms and the distal 3′-UTR regions using Primer3 (http://Frodo.wi.mit.edu). PCR was performed with Platinum™ SuperFi II DNA Polymerase (Invitrogen, 12361050). PCR conditions: 30 s at 98°C, 15 s at 60°C, and 30 s at 72°C. PCR products were analyzed on a 2% agarose gel. The primer sequences used are listed in Supplementary Table S1.

## Results

### Isoforms-Level Expression Provides Extra Information to Distinguish Oocyte Stages

We generated two batches of single oocyte RNA-seq data from 16 oocytes at the GV stage, 15 at the MI stage and six at the MII stage. For most of the 37 oocytes, we sequenced each with more than 15 million paired-end reads (Supplementary Figure S1A), and the reads were then mapped to the mouse reference genome (mm10) and quantified against GENCODE gene models (version VM20) at both the gene and isoform levels. Principal component analysis (PCA) revealed a strong batch effect of our data not only at the gene level (Supplementary Figures S1B,C) but also the isoform level (Supplementary Figures S1D,E), which dominated the first principal component (PC 1). To correct the batch effect in our data we tried a few batch-effect correction algorithms, and it turned out that Combat (Johnson et al., 2007) performed the best with respect to oocyte clustering results (see below), which is consistent with previous reports (Chen et al., 2020; Tran et al., 2020).

**FIGURE 1:**
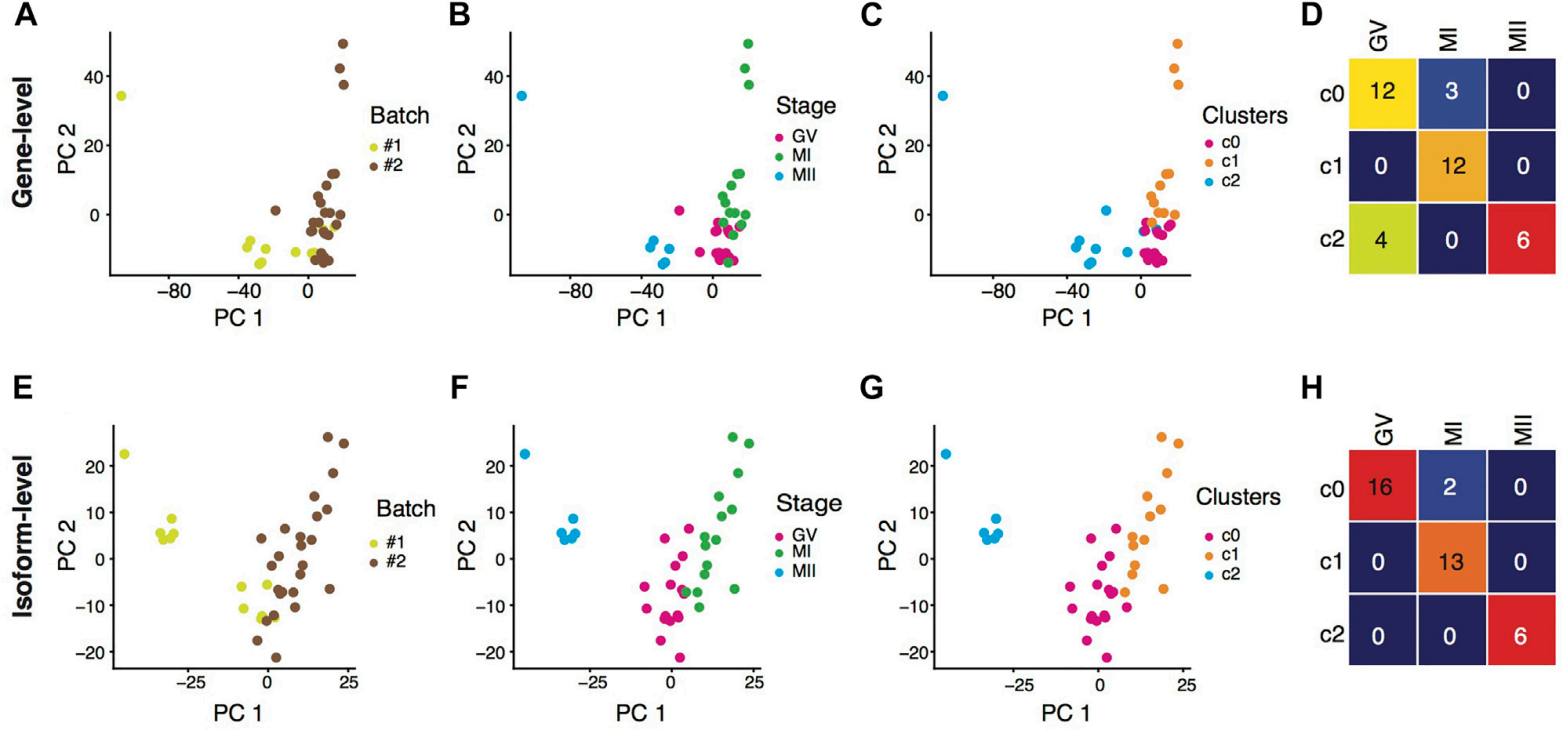
Isoform expression outperformed gene expression in clustering oocytes. **(A–C)** PCA visualization of individual oocytes based on batch-effect corrected gene-level expression, colored by batches **(A)**, phenotypic stages **(B)**, and expression clustering results **(C)**. **(D)** Heatmap showing the contingency table between phenotypic stages and clustering results based on batch-effect corrected gene-level expression. **(E–G)** PCA visualization of individual oocytes based on batch-effect corrected isoform-level expression, colored by batches **(E)**, phenotypic stages **(F)**, and expression clustering results **(G)**. **(H)** Heatmap showing the contingency table between phenotypic stages and clustering results based on batch-effect corrected isoform-level expression.

After batch-effect correction, we took the gene-level (Figures 1A,B) and the isoform-level (Figures 1E,F) expression profiles, respectively, as the input for oocyte clustering analysis. Although there was heterogeneity among transcriptomes of individual oocytes, oocytes at the MII stage were different dramatically from oocytes at the GV stages after entering the meiotic resumption process, featured by many unnecessary transcripts starting to degrade (Wu and Dean, 2020; Zhao et al., 2020). Therefore, we downgraded the unsupervised clustering results which did not entirely separate GV and MII oocytes into distinct clusters. In terms of their transcriptomes, oocytes at the GV and the MI stage were more similar, as shown in a previous study in human oocyte maturation that there were hardly any differentially expressed genes (DEGs) between the GV and MI stages, but a similar number of DEGs between MI vs. MII and between GV vs. MII (Yu et al., 2020). With this knowledge, we did not consider the clear segregation of GV and MI oocytes as a criterion to evaluate the clustering results. According the above criteria, it is clear that the clustering result from gene-level analysis (Figures 1C,D) was less favorable than the result from isoform-level analysis. Hence, we conclude that rather than aggregating isoforms into gene loci, there is extra transcriptomic information contained in individual isoforms, useful for better understanding the dynamics in the transition from meiotic arrest to resumption during mouse oocyte maturation.

### Differentially Expressed Genes and Isoforms During Oocyte Maturation

Since we observed an obvious batch effect in our data (Supplementary Figure S1B,D), we applied two strategies including comparison within one batch and application of a covariant model to account for the batch factor, to compare the transcriptomes of GV vs. MI oocytes (Supplementary Table S2) and GV vs. MII oocytes (Supplementary Table S3). We found that different strategies gave similar results, and consistently transcriptomes of GV vs. MII were more divergent than that of GV vs. MI. Hence, we then focused on the comparison of GV vs. MII based on the first batch of our data, which were generated from 6 GV oocytes and six MII oocytes. At the gene level, there were 1,565 genes specifically expressed at the GV stage, compared to 934 genes uniquely expressed at the MII stage (Supplementary Figure S2A). This is consistent with previous results that there were more GV-specific genes than MII-specific ones (Zhao et al., 2020), as transcriptional silence got started during the GV to MII transition. Similar phenomenon was observed at the isoform level (Supplementary Figure S2B). Of note, we considered here only the relative abundance of genes and transcripts, so that we were able to detect genes and transcripts of absolutely lower abundance at the MII stage. Taking this into account, it is very likely that the number of GV-specific genes was understated while the number of MII-specific genes was overstated.

**FIGURE 2:**
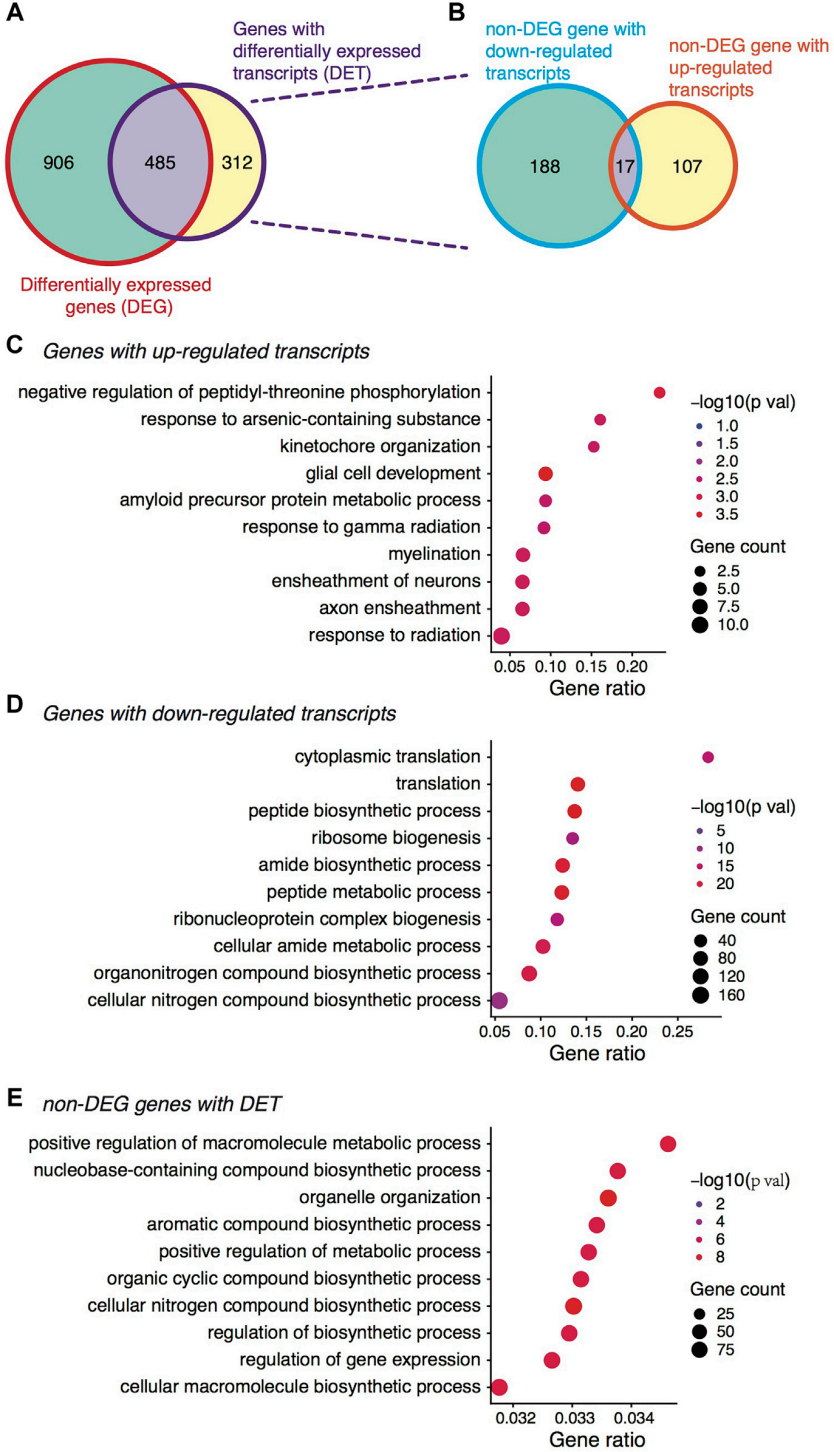
Differential expression analysis between the GV and MII oocytes. **(A)** Venn diagram showing the overlap between DEGs and genes with DETs. **(B)** Out of the 312 non-DEG genes with DETs, Venn diagram showing the distribution of genes with up- and down-regulated transcripts. **(C)** Gene ontology (GO) term enrichment analysis of gene with up-regulated transcripts. **(D)** GO term enrichment analysis of gene with down-regulated transcripts. **(E)** GO term enrichment analysis of the non-DEG genes yet with DETs.

We then analyzed the expression profiles between the two stages for differentially expressed genes (DEGs) and transcripts (DETs). Again, we considered here the relative expression changes albeit a global shift towards downregulation was observed during the transition (Wu and Dean, 2020). With the *edgeR* algorithm, we detected 591 genes exhibiting relatively higher expression at the MII stage whereas 800 genes showing lower expression (Supplementary Figure S2C). At the isoform level, 220 transcripts were upregulated while 660 were downregulated (Supplementary Figure S2D). Interestingly, when assigning the DETs to their host genes, we found more than one third of these genes (39.1% or 312 genes) not discovered by DEG analysis (Figure 2A), demonstrating again the additional information provided by isoform-level analysis. Of the 312 genes, 17 genes had both transcripts upregulated and downregulated at the MII stage (Figure 2B). In such cases, the expression differences at the gene level could be canceled out, thus becoming undetectable. There were also quite a number of genes (906) only showing differential expression at the gene level but not the isoform level (Figure 2A), possibly due to two different reasons. First, aggregating isoforms that exhibited weak differences made the differences at the gene level much stronger; second, large uncertainty existed in the isoform expression inference based on short sequencing reads, which resulted in larger variability and attenuated statistical power. Thus, when isoform expression quantification became more accurate, the number of genes showing only differences at the gene level should be reduced.

Gene ontology (GO) enrichment analysis showed that genes with upregulated transcripts or upregulated genes at the MII stage were mainly related to neuronal peptides-mediated processes (Figure 2C and Supplementary Figure S2E), which have been shown in low animals to be related to oocyte maturation (Kato et al., 2009; Quiroga Artigas et al., 2020). By contrast, the genes with downregulated transcripts or downregulated genes were enriched in functions related to ribosome biogenesis and translation (Figure 2D and Supplementary Figure S2F), again consistent with previous observations (Zhao et al., 2020). Intriguingly, the 312 genes unidentifiable by DEG analysis but with DETs were functionally related to macromolecule metabolic process and regulation of gene expression (Figure 2E), coinciding with a recent study on porcine oocytes *in vitro* maturation where the authors found such genes were downregulated during maturation (Brazert et al., 2020). Organelle organization was another enriched GO term for the 312 genes (Figure 2E), and the role of oocyte organelle has been shown to related to oocyte developmental competence and therefore oocyte quality (Reader et al., 2017). Importantly, these functionally critical genes were only identified by isoform-level analysis, further highlighting the usefulness of considering isoform-specific changes during mouse oocyte maturation.

### Isoform Changes Predominantly Contributed by Alternative 3′-UTR Usage

We next sought to dissect the alternative RNA processing events that contributed to isoform changes between GV and MII oocytes. According to a benchmarking study (Mehmood et al., 2020) and our experience (He et al., 2021b), we first adopted *SUPPA2* (Trincado et al., 2018) and *rMATS* (Shen et al., 2014) for comparison of annotated alternative events. *SUPPA2* investigated seven types of alternative events including skipped exons, retained introns, mutually exclusive exons, alternative 5′ or 3′ splice sites, and alternative first or last exons; however tandem UTRs were not included. In our data, *SUPPA2* identified from the whole transcriptome only four alternative events differentially used between GV and MII oocytes at false discovery rate (FDR) of 0.05 (Supplementary Figure S3A). While *SUPPA2* might be over-conservative, *rMATS* did not identify many alternative events of differential usage between the two stages, either (Supplementary Figure S3B), partially because *rMATS* only focused on internal alternative splicing events, so that entirely ignored structural differences at both ends of a transcript.

**FIGURE 3:**
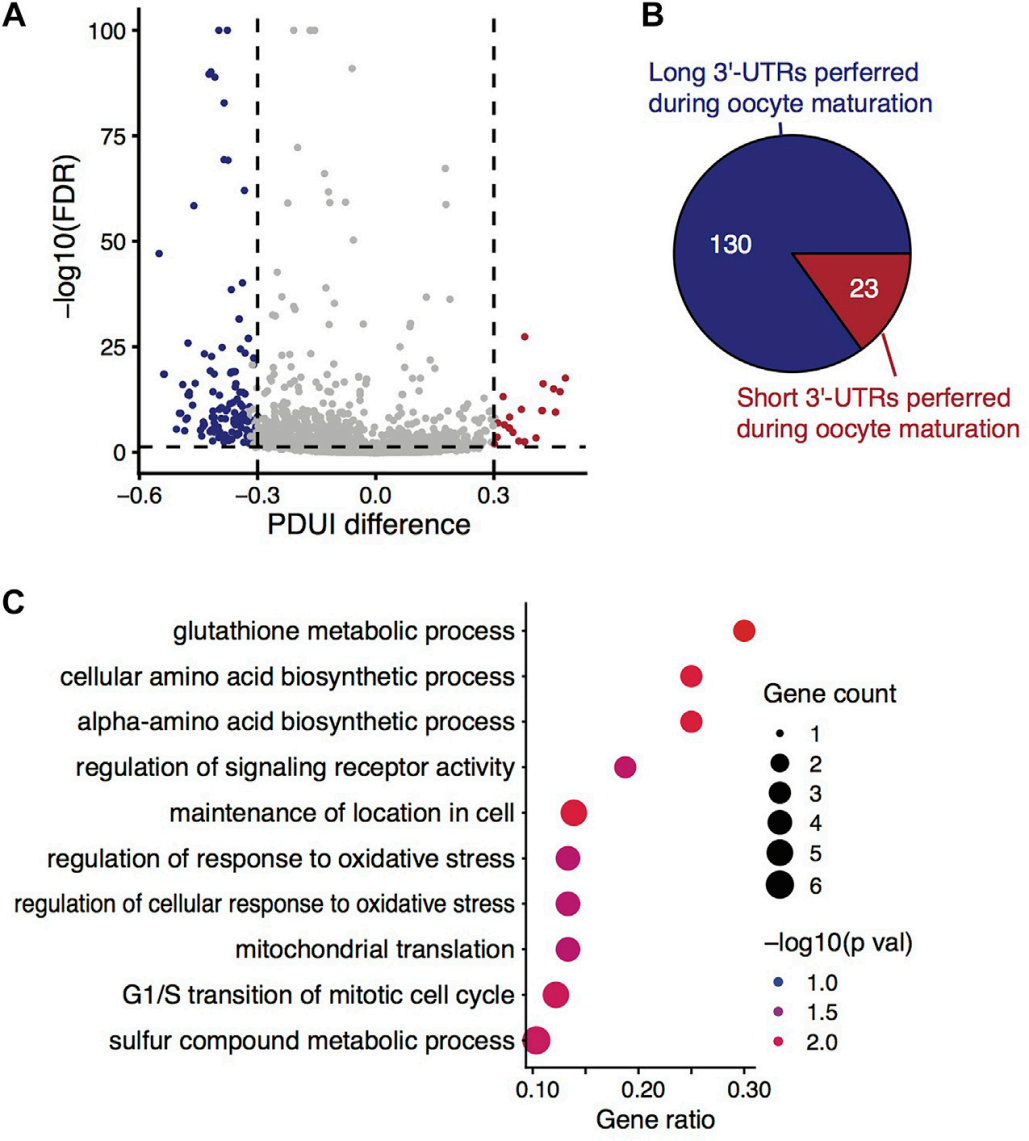
Alternative 3′-UTR isoform switches during mouse oocyte maturation. **(A)** Volcano plot showing the differences in percentage of distal poly(A) site usage index (PDUI) between isoform pairs against the associated statistical significance level (FDR). **(B)** Pie chart demonstrating the asymmetric 3′-UTR isoform switches towards longer UTRs along with oocyte maturation. **(C)** GO term enrichment analysis of genes exhibiting significantly alternative usage of 3′-UTR isoforms during the oocyte maturation.

Given that the isoform-level analysis indeed provided useful information in distinguishing the two stages from the above analyses, we wondered which alternative events could be the main reasons responsible for the distinct isoform-level differences. By genome-browser enabled visualization investigation of reads mapped to the genome, we found many genes exhibited differences in read coverage near the 3′-ends. According to it, we finally utilized *DaPars* (Xia et al., 2014) for analysis of alternative polyadenylation sites (PASs) regardless of gene structure annotation. Not surprisingly, *DaPars* identified 153 events happening in 128 genes at its default significance level (Figure 3A), which we regarded as a set of very stringent criteria (i.e., FDR ≤0.05, fold change of PDUI ≥1.5, and absolute differences of PDUI ≥0.3). When losing the criteria to FDR ≤0.05 and the absolute differences of PDUI ≥0.15, we found 663 (10.7%) significant alternative isoform pairs differentially used in the two stages out of 6,213 tested pairs, or 512 (13.7%) genes harboring such significant events out of 3,810 tested genes (Supplementary Table S4). Hence, we concluded that the main source of isoform-level expression changes during oocyte maturation was the alternative usage of 3′-UTRs, or more specifically, the alternative usage of PASs.

### Alternative 3′-UTR Isoforms Used Between GV and MII Oocytes

We then asked whether there was a tendency of 3′-UTR length changes among these genes exhibiting alternative usage of 3′-UTR isoform during mouse oocyte maturation. By looking into the direction of PDUI values, we found a clear preference (85.0%) towards maintaining longer 3′-UTR isoforms against degradation during the process (Figure 3B). Even at the loosed PDUI threshold, 68.3% (478 vs. 222) of the significant isoform pairs showed the preference towards longer ones.

Regardless of the length preference, GO enrichment analysis revealed the genes with 3′-UTR isoform switches during oocyte maturation were functionally linked to glutathione metabolic process, cellular response to oxidative stress, maintenance of location in cells, etc. (Figure 3C). It has been reported that the cellular concentration of glutathione changes during oocyte maturation (Luberda, 2005), and glutathione plays certain roles in protecting oocytes against oxidative damage (Brad et al., 2003) and in maintaining the meiotic spindle morphology of the oocytes (Zuelke et al., 1997). Similarly, cellular location maintenance or relocation/redistribution of particular organelles (e.g., mitochondria) in oocytes was closely related to oocyte quality (Mao et al., 2014). Therefore, the genes with 3′-UTR isoform composition changes are of functional importance in safeguarding the process of oocyte maturation.

**FIGURE 4:**
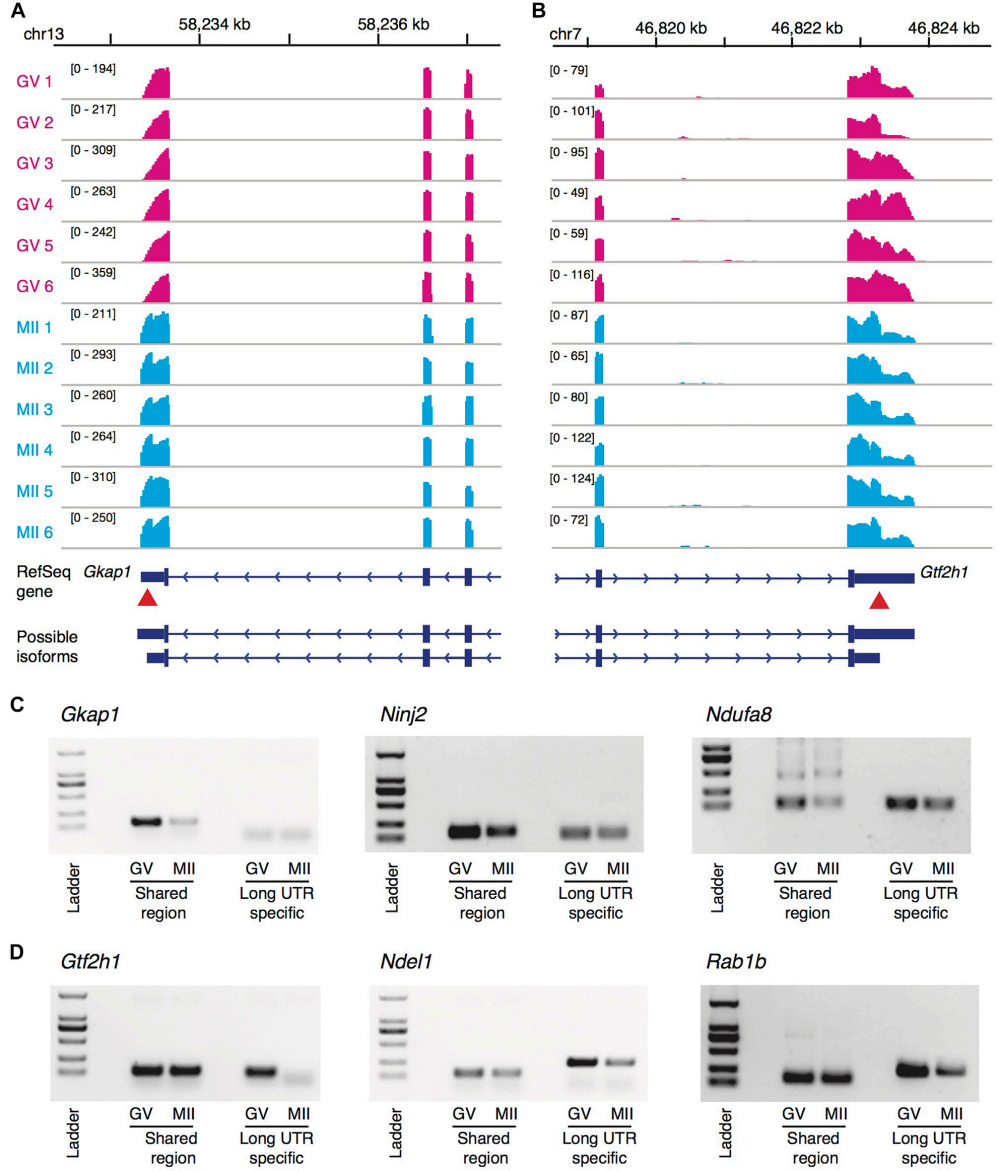
Genome-browser visualization and experiment validation of 3′-UTR isoform switches. **(A,B)** Genome-browser visualization of the genes *Gkap1* **(A)** and *Gtf2h1* **(B)**. In each subplot, showing from top to bottom are genomic coordinates, RNA-seq read coverage of 6 GV oocytes (red), RNA-seq read coverage of six MII oocytes (blue), the RefSeq gene annotation, and possible isoforms with alternative polyadenylation sites. The red arrows point out the approximate location of the proximal polyadenylation sites (i.e., the switch points on read coverage between the two stages). **(C)** Validation of the genes *Gkap1*, *Ninj2*, and *Ndufa8* with longer 3′-UTR isoforms preferred during the oocyte maturation by independent experiments. Taking the shared region as a reference, the long UTR-specific region either showed weaker bands at the GV stage (*Gkap1* and *Ninj2*) or a stronger band at the MII stage (*Ndufa8*). **(D)** Similar to **(C)**, but validation of genes *Gtf2h1*, *Ndel1*, and *Rab1b*, which showed the opposite trend in length of the 3′-UTR isoform usage.

Of the 128 genes at the top significance, we randomly picked up six genes for independent experimental validation, including three genes that tended to use longer 3′-UTR isoforms during the maturation and the other three with the opposite tendency. In our single-oocyte RNA-seq data, genes *Gkap1*, *Ninj2*, and *Ndufa8* tended to switch to the longer 3′-UTR isoforms during the GV to MII transition (Figure 4A; Supplementary Figures S4A,B), which was validated by the independent experiments (Figure 4C): Taking the shared region as a reference, the long UTR-specific region either showed weaker bands at the GV stage (*Gkap1* and *Ninj2*) or a stronger band at the MII stage (*Ndufa8*). In the contrary, genes *Gtf2h1*, *Ndel1*, and *Rab1b* exhibited a tendency to preferably express shorter 3′-UTR isoforms at the MII stage (Figure 4B; Supplementary Figures S4C,D), and were also clearly recapitulated by the validation experiments (Figure 4D).

## Discussion

In this study, we showcased the importance of isoform-level expression analysis when applying RNA-seq or similar technologies to developmental and reproductive biology, such as in the maturing mouse oocytes. Along with the line that isoform expression quantification provided essential information in more accurately clustering oocytes at different stages, we revealed pervasive alternative 3′-UTR isoform usage during the GV to MII oocyte transition. Although most of the engaged genes tended to have their longer 3′-UTR isoforms more stable, resulting in higher abundance at the MII stage, GO enrichment analysis highlighted the functional importance of these genes regardless of the direction of the changes.

The isoform-level expression changes could serve as fine-tune regulation of the gene-level abundance during the dynamic process. Specifically, since transcription became silenced after entering the meiotic resumption, translational regulation would play a more predominant role in controlling the protein concentration. Reselecting *cis*-regulatory elements [such as RNA binding protein binding sites (Yamashita and Takeuchi, 2017) and miRNA target sites (Oliveto et al., 2017)] residing in the UTR regions *via* degrading particular transcripts might be, however, regarded as a global machinery during the transition, to accommodate the transcriptionally silent mode for a certain time period.

In addition, cytoplasmic polyadenylation would be happening after transcriptional silence in maturing oocytes (Reyes and Ross, 2016), which also contributed to translational regulation for protein synthesis (Radford et al., 2008). Therefore, studying the regulation coordination or synergistic regulation of 3′-UTR isoform selection and cytoplasmic polyadenylation of particular transcripts would uncover the detailed mechanisms in shaping the protein landscape of MII oocytes, and also preparing the sufficient molecular materials for upcoming fertilization and early embryonic development, until zygotic genome activation at approximately the two-cell stage for mice and eight-cell stage for human.

From the technical aspect, the present study still adopted the short-read sequencing technology, which could not usually generate sequencing reads longer than 300 nt. This is the exclusive reason for having to infer the isoform-level expression, instead of direct quantification by counting reads. Due to the identifiability of isoform expression inference (Hiller et al., 2009) and large uncertainty introduced in the process (Jiang and Wong, 2009), isoform-level analysis did not show clear advantages over gene-level analysis in expression changes between the two stages. With the rapid development and wide acceptance of the third-generation long-read sequencing technologies (including PacBio and Nanopore), full-length transcriptome sequencing (Wang et al., 2019; Hu et al., 2020) would offer more direct information for isoform-level analysis. Most recently, methods have been developed to sequence poly(A)-tail inclusive full-length transcriptome using the PacBio platform (Legnini et al., 2019; Liu et al., 2019), which would be extremely helpful for studying the detailed molecular mechanisms during the dynamic process of oocyte maturation.

## Data Availability

The datasets presented in this study can be found in online repositories. The names of the repository/repositories and accession number(s) can be found below: https://www.ncbi.nlm.nih.gov/geo/query/acc.cgi?acc=GSE178836.

## Fundings

This study was funded in part by the National Natural Science Foundation of China (Project No. 32100681, 82101699, and 32170742), the China Postdoctoral Science Foundation (General Program No. 2019M651894), the Nanjing Medical University Research Start-up Fund (Project No. NMUR2020009), and the Opening Fund of Key Laboratory of the Diagnosis and Treatment Research of Reproductive Disorders of Zhejiang Province (No. 2018002).
