# Supplementary material for: Pervasive 3′-UTR Isoform Switches During Mouse Oocyte Maturation
Source: Front Mol Biosci. 2021 Oct 18;8:727614. doi: 10.3389/fmolb.2021.727614 (PMC8558312; doi:10.3389/fmolb.2021.727614)
Supplement: Supplementary file 3 [file Table1.pdf]

# *Supplementary Tables*

### Pervasive 3'-UTR isoform switches during mouse oocyte maturation

**Yuanlin He, Qiuzhen Chen, Jing Zhang, Jing Yu, Meng Xia, Xi Wang**

**Supplementary Table 1. Oligonucleotide primers used in this study.**

| Name | Forward primer (5'-3') | Reverse primer (5'-3') | Amplicon Size (bp) |
|---|---|---|---|
| Gkap1_shared | GAAGGGGAAAAGGAACTTCG | GCAGTTTAGCAGTCGCACAG | 123 |
| Gkap1_distal | CAGAATACCAGCAGAACTTGATT | TCATAAGAAGTTTGGACAGAAAACA | 50 |
| Ninj2_shared | TCAGCCTTTGTCCCTGACTT | TCTGGTGCTGTGGACAAGAG | 105 |
| Ninj2_distal | CTGCACTGCCTTCTTTTCCT | GCAGAAAGGGAACATTGAGG | 50 |
| Ndufa8_shared | ACTCAAGAGCAAGGCCAGAG | GGACCGTCAACCCATCTCTA | 109 |
| Ndufa8_distal | GTGCCGATCTCGTGTTCTTT | AGAATAACTGCCAAGTCACATAAA | 75 |
| Gtf2h1_shared | CTCTGGAAACCTGGCTGAAG | ACAGCATCAGCTCTGGGAGT | 87 |
| Gtf2h1_distal | GTCCCCAAGTAGAAGGCACA | CAGCATCAGTCTCCTGACGA | 69 |
| Ndel1_shared | AAATGGCTTTGATCCAGCTC | CACACACTGAGAGGCAGCAT | 87 |
| Ndel1_distal | CGTTGGTTTCACATGATTGC | TGAGCACTGAAGATGCTTGG | 227 |
| Rab1b_shared | GACAATGGCTGCAGAGATCA | TGCTGTCGATCTTCAGGTTG | 83 |
| Rab1b_distal | AGCTGCGGTTAGGTCTTGAG | TGGCAGAGTGAGGGTTAAGG | 219 |

**Supplementary Table 2. Differentially expressed genes and transcript isoforms between GV and MI oocytes.**

**Supplementary Table 3. Differentially expressed genes and transcript isoforms between GV and MII oocytes.**

**Supplementary Table 4. Alternative 3'-UTR isoform usage between GV and MII oocytes inferred by *Dapars*.**

*(See separate Excel files for Supplementary Tables 2 - 4.)*
