# Supplementary material for: Pervasive 3′-UTR Isoform Switches During Mouse Oocyte Maturation
Source: Front Mol Biosci. 2021 Oct 18;8:727614. doi: 10.3389/fmolb.2021.727614 (PMC8558312; doi:10.3389/fmolb.2021.727614)
Supplement: Supplementary file 4 [file Presentation1.pdf]

Supplementary Figures

Pervasive 3'-UTR isoform switches during mouse oocyte maturation

Yuanlin He, Qiuzhen Chen, Jing Zhang, Jing Yu, Meng Xia, Xi Wang

This file contains Supplementary Figures 1 – 4.

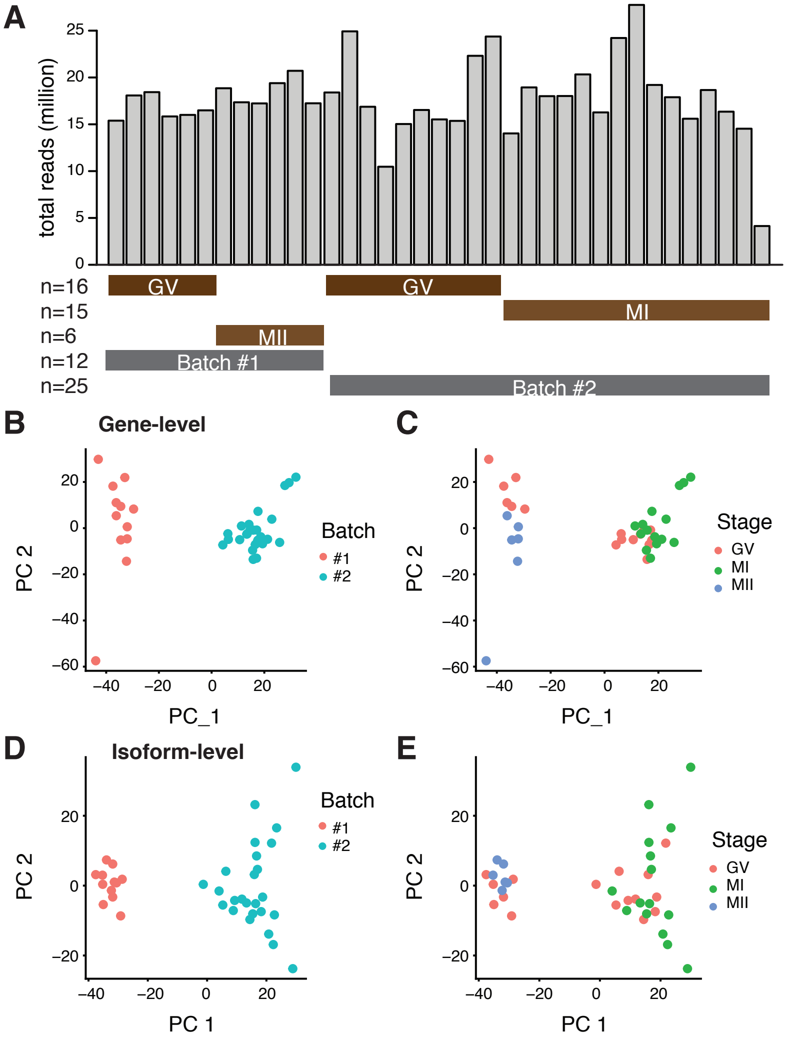

Supplementary Figure 1. Single-oocyte transcriptome sequencing experiment and analysis. (A) The overview of this study. Bar plots showing the amount of data generated in this study. (B-C) PCA visualization of individual oocytes based on gene-level expression (without batch-effect correction), colored by batches (B), and phenotypic stages (C). (D-E) PCA visualization of individual oocytes based on isoform-level expression (without batch-effect correction), colored by batches (D), and phenotypic stages (E).

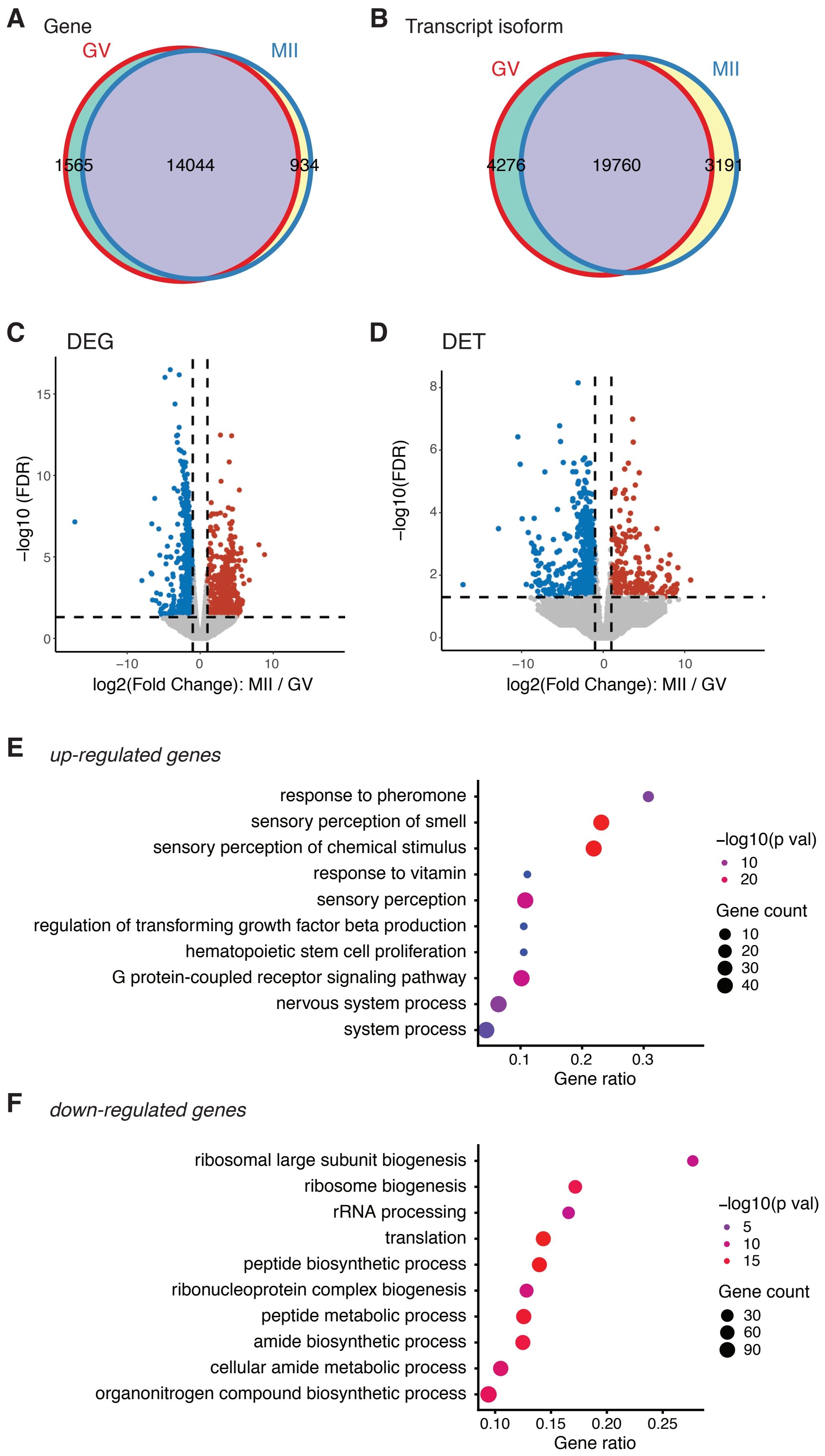
A
Gene
GV
MII
1565
14044
934
B
Transcript isoform
GV
MII
4276
19760
3191
C
DEG
−log10 (FDR)
15
10
5
0
−10
0
10
log2(Fold Change): MII / GV
D
DET
−log10(FDR)
8
6
4
2
0
−10
0
10
log2(Fold Change): MII / GV
E
up-regulated genes
response to pheromone
sensory perception of smell
sensory perception of chemical stimulus
response to vitamin
sensory perception
regulation of transforming growth factor beta production
hematopoietic stem cell proliferation
G protein-coupled receptor signaling pathway
nervous system process
system process
0.1
0.2
0.3
Gene ratio
−log10(p val)
10
20
Gene count
10
20
30
40
F
down-regulated genes
ribosomal large subunit biogenesis
ribosome biogenesis
rRNA processing
translation
peptide biosynthetic process
ribonucleoprotein complex biogenesis
peptide metabolic process
amide biosynthetic process
cellular amide metabolic process
organonitrogen compound biosynthetic process
0.10
0.15
0.20
0.25
Gene ratio
−log10(p val)
5
10
15
Gene count
30
60
90

Supplementary Figure 2. Differential expression analysis between oocytes at the GV and MII stages. (A) Venn diagram showing the expressed genes between the two stages. (B) Venn diagram showing the expressed transcripts between the two stages. (C) Volcano plot showing differentially expressed genes between GV and MII oocytes using *edgeR*. (D) Volcano plot showing differentially expressed transcript isoforms between GV and MII oocytes using *edgeR*. (E) Gene ontology (GO) term enrichment analysis of up-regulated genes during oocyte maturation. (F) GO term enrichment analysis of down-regulated genes during oocyte maturation.

A *SUPPA2*

Gene	Alternative Event Type	PSI difference	FDR
Ccdc82	SE	0.541	0
Cdk20	RI	0.735	0
Psen1	AF	0.441	0
Arpp19	A5	0.486	0

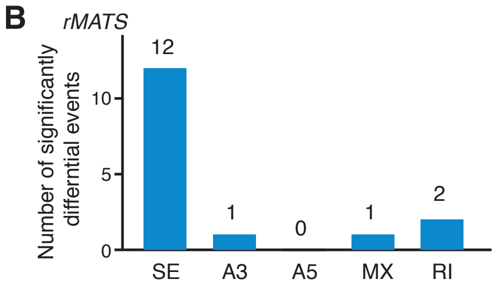

Supplementary Figure 3. Differential splicing events detected by SUPPA2 and rMATS between GV and MII oocytes. (A) The only four alternative events differentially used between GV and MII oocytes detected by SUPPA2. (B) Bar plots showing the number of differentially used alternative events between the two stages, identified by rMATS.

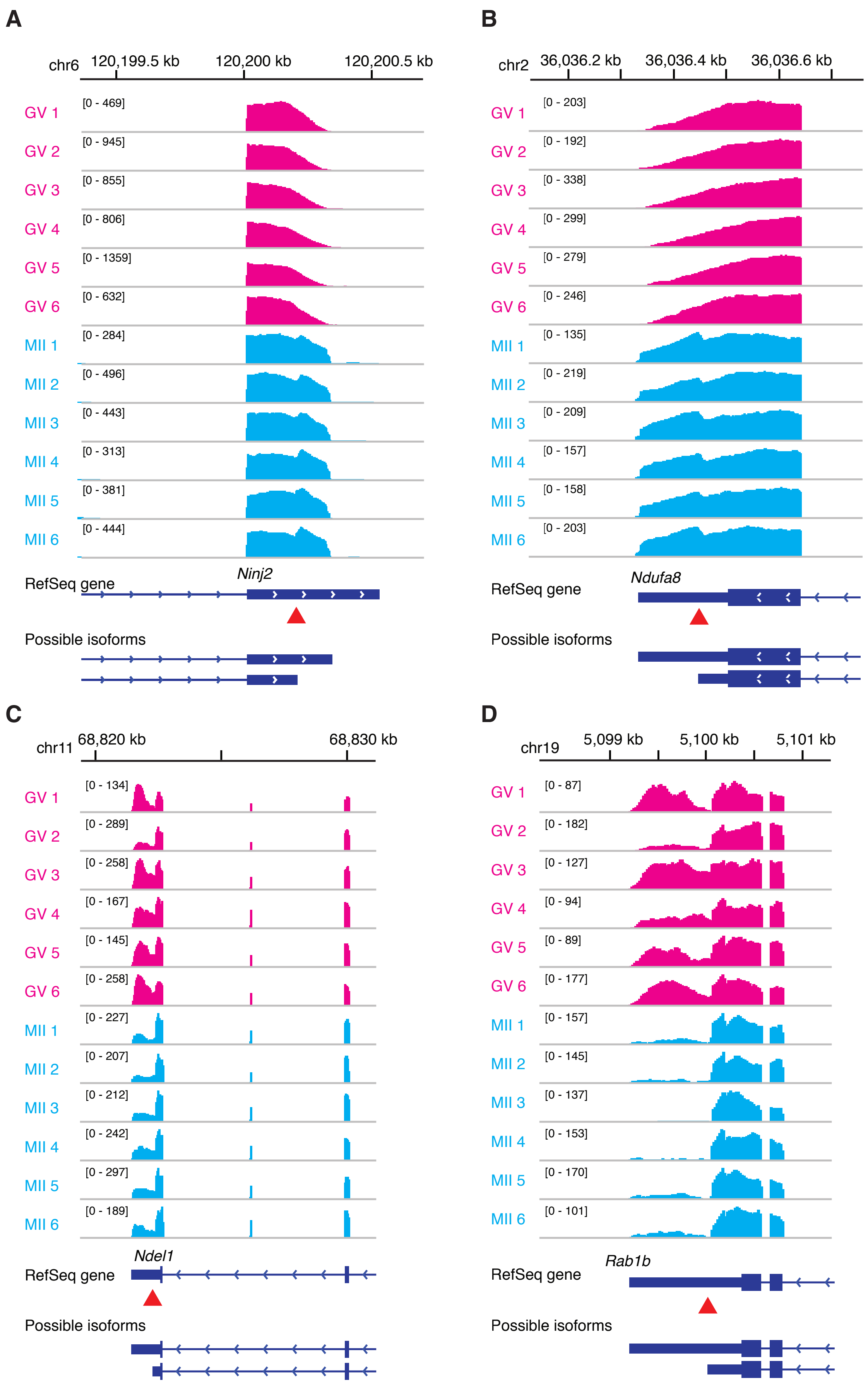
A
chr6
120,199.5 kb
120,200 kb
120,200.5 kb
GV 1
GV 2
GV 3
GV 4
GV 5
GV 6
MII 1
MII 2
MII 3
MII 4
MII 5
MII 6
[0 - 469]
[0 - 945]
[0 - 855]
[0 - 806]
[0 - 1359]
[0 - 632]
[0 - 284]
[0 - 496]
[0 - 443]
[0 - 313]
[0 - 381]
[0 - 444]
RefSeq gene
Ninj2
Possible isoforms
B
chr2
36,036.2 kb
36,036.4 kb
36,036.6 kb
[0 - 203]
[0 - 192]
[0 - 338]
[0 - 299]
[0 - 279]
[0 - 246]
[0 - 135]
[0 - 219]
[0 - 209]
[0 - 157]
[0 - 158]
[0 - 203]
Ndufa8
C
chr11
68,820 kb
68,830 kb
[0 - 134]
[0 - 289]
[0 - 258]
[0 - 167]
[0 - 145]
[0 - 258]
[0 - 227]
[0 - 207]
[0 - 212]
[0 - 242]
[0 - 297]
[0 - 189]
Ndel1
D
chr19
5,099 kb
5,100 kb
5,101 kb
[0 - 87]
[0 - 182]
[0 - 127]
[0 - 94]
[0 - 89]
[0 - 177]
[0 - 157]
[0 - 145]
[0 - 137]
[0 - 153]
[0 - 170]
[0 - 101]
Rab1b

Supplementary Figure 4. Genome-browser visualization of 3'-UTR isoform switches in additional genes. The visualized genes are *Ninj2* (A), *Ndufa8* (B), *Ndel1* (C), and *Rab1b* (D). In each subplot, showing from top to bottom are genomic coordinates, RNA-seq read coverage of six GV oocytes (red), RNA-seq read coverage of six MII oocytes (blue), the RefSeq gene annotation, and possible isoforms with alternative polyadenylation sites. The red arrows point out the approximate location of the proximal polyadenylation sites (i.e. the switch points on read coverage between the two stages).
